# Preoperative EUS–guided biopsy does not affect survival in patients with pancreatic cancer: A nationwide cohort study

**DOI:** 10.1097/eus.0000000000000156

**Published:** 2025-12-15

**Authors:** Bojan Kovacevic, Claus Fristrup, Carsten P. Hansen, Michael B. Mortensen, Frank Mortensen, Jakob Kirkegård, Mogens T. Stender, Sönke Detlefsen, Peter Vilmann

**Affiliations:** 1 Department of Gastrointestinal and Hepatic Diseases, Copenhagen University Hospital – Herlev and Gentofte, Denmark; 2 Danish Pancreatic Cancer Group, Odense University Hospital, Denmark; 3 Department of Surgical Gastroenterology, Odense University Hospital, Denmark; 4 Department of Digestive Diseases, Transplantation and General Surgery, Copenhagen University Hospital – Rigshospitalet, Denmark; 5 Department of Clinical Research, Faculty of Health Sciences, University of Southern Denmark, Denmark; 6 Department of Surgical Gastroenterology, Aarhus University Hospital, Denmark; 7 Department of Surgical Gastroenterology, Aalborg University Hospital, Denmark; 8 Department of Pathology, Odense University Hospital, Denmark; 9 Department of Clinical Medicine, University of Copenhagen, Denmark.

**Keywords:** EUS-FNA, EUS-FNB, PDAC, Survival, Pancreatic cancer, Biopsy

## Abstract

**Background and Objectives:**

Pancreatic cancer is a highly aggressive malignancy with poor prognosis. Surgery is the only curative treatment, but it carries a significant risk of morbidity. The role of preoperative EUS-guided biopsy (EUS-B) in up-front resectable patients has been a matter of debate, with some centers strongly advocating its use, whereas others limit it to indeterminate cases.

We aimed to examine whether preoperative EUS–guided biopsy (EUS-B) has an impact on overall survival (OS) using nationwide data.

**Methods:**

The data of patients who underwent curatively intended surgical resection for pancreatic cancer were retrieved from the prospectively maintained national Danish Pancreatic Cancer Group database. Associations between preoperative EUS-B and survival were evaluated using Kaplan-Meier plots and univariable and multivariable Cox proportional hazards models. OS was examined in the overall cohort and in a propensity score–matched subgroup, where EUS-B cases were matched to controls in a 1:2 ratio.

**Results:**

Between 2011 and 2023, 1889 patients who underwent surgery for pancreatic cancer constituted the overall cohort. The mean age was 67.4 (SD, 9.1) years, and 53.5% were male. The median overall survival was 28.5 months in the EUS-B group compared to 26.7 months in the non–EUS-B group (HR, 1.07; 95% CI, 0.91–1.26; *P* = 0.411). In the propensity score–matched subgroup (*n* = 582), the median survival time was 28.6 months (EUS-B group) and 24.7 months (non–EUS-B group; HR, 1.04; 95% CI, 0.84–1.29; *P* = 0.691).

**Conclusions:**

Preoperative EUS-B did not influence survival in patients with pancreatic cancer in this nationwide retrospective study.

## INTRODUCTION

Despite advances in cancer diagnosis and treatment, pancreatic cancer remains one of the most aggressive malignancies with a poor prognosis. Radical surgery offers the only potential for long-term survival; however, accurate preoperative staging and diagnosis are crucial for optimal treatment planning. EUS–guided biopsy (EUS-B), which was first performed several decades ago, is now a widely used diagnostic modality for histological or cytological diagnosis of pancreatic lesions.^[1]^ Although EUS-B is generally regarded as safe and effective, concerns have been raised about its potential impact on survival due to the theoretical risk of tumor cell dissemination along the needle tract. Although this risk is lower than that of percutaneous biopsies, it has been reported in few cases.^[2]^ Additionally, there is a small risk of adverse events, which could prolong the time until treatment begins, as well as the issue of false-negative biopsies, both of which support the argument for recommending surgery without prior histopathological confirmation. However, this remains a matter of debate, with global and regional variations in the use of preoperative EUS-B. The ESMO guidelines do not recommend routine preoperative biopsy of pancreatic lesions, reserving this technique for indeterminate cases.^[3]^ In contrast, the Clinical Practice Guidelines for Pancreatic Cancer published by the Japan Pancreas Society strongly endorse preoperative histopathological diagnosis using EUS-B.^[4]^ The effect of neoadjuvant chemotherapy (NACT) in up-front resectable pancreatic cancer is still debated, but NACT will, in most, cases require prior pathological confirmation of malignancy.

The implications of EUS-B for survival have been addressed in a few retrospective studies. However, existing research is often limited by small sample sizes, single-center designs, and inadequate adjustments for confounders.^[5,6]^ Using a national, prospectively maintained database, we aimed to examine overall survival following EUS-B in a large cohort of patients treated at 4 tertiary surgical centers across Denmark.

## METHODS

This retrospective, nationwide cohort study examined the effects of preoperative EUS-B on overall survival in patients with pancreatic carcinoma. The prospectively maintained Danish Pancreatic Cancer Group (DPCG) database was queried for patients who underwent radical surgical resection for pancreatic cancer between 2011 and 2023. The database contains high-quality data provided by all 4 tertiary centers involved in the surgical treatment of pancreatic cancer in Denmark, with estimated completeness of data capture exceeding 95%. Furthermore, the database is directly coupled with the Danish Civil Registration System (CPR), ensuring reliable data on mortality and emigration. Patients with periampullary and neuroendocrine tumors were excluded from this study. The use of preoperative EUS-B varies among the 4 treatment centers and is influenced by both availability and local tradition, thus creating a unique form of pseudo-randomization at the national level.

The DPCG database does not contain data on recurrence, nor does it provide the cause of mortality, which is why overall survival was chosen as a primary outcome, defined as the time between surgery and death or end of follow-up. Patients were censored upon emigration or at the end of January 2024. For tumor staging, both the eighth edition of the American Joint Committee on Cancer (AJCC) and TNM staging system were utilized based on postoperative histopathological diagnosis. The primary outcome was analyzed in the whole cohort, as well as in a subset of patients forming a propensity score–matched cohort. The study was conducted in accordance with the principles of the Declaration of Helsinki. The use of register data followed the General Data Protection Regulation of the European Union and the Danish Data Protection Agency guidelines and was consented by The Regional Committee on Health Research Ethics for Southern Denmark (no. 23/18843).

### Propensity score matching

To obtain estimates that were as unbiased as possible, only treatment-naïve patients who underwent EUS-B were matched to non–EUS-B patients at a ratio of 1:2. This matching ratio was chosen to maximize statistical power while maintaining good covariate balance due to the limited number of EUS-B cases compared to non–EUS-B. Matching was based on propensity scores using the nearest-neighbor matching method. Propensity scores were calculated using the following variables: age, sex, Charlson comorbidity index (CCI), AJCC tumor stage, type of anatomical resection, vessel resection, treatment center, and adjuvant oncological therapy. The results of the matching were evaluated by examining the baseline characteristics and the standardized mean differences between the two groups.

### Statistical analyses

The baseline characteristics of the two groups were summarized by means with standard deviations, medians with range, or absolute numbers and proportions, when appropriate. Associations between EUS-B and overall survival were evaluated using Kaplan-Meier plots and univariable and multivariable Cox proportional hazards models. Variables with a 2-sided *P* value <0.1 in univariable analysis were included in the multivariable model. The final model was therefore adjusted for age, sex, CCI, tumor stage, type of resection (accounting for tumor location and surgical approach), and adjuvant therapy. Potential multicollinearity was assessed using the variance inflation factor (VIF). Proportional hazard assumptions were tested using the Grambsch-Therneau test and by examining Schoenfeld residuals. Effect sizes were expressed as hazard ratios, median survival times, and Kaplan-Meier plots. When examining the effects of adjuvant therapy, a time-varying exposure model was employed to account for the possible immortal time bias. Patients were considered to be nonexposed to adjuvant treatment from the date of surgery until initiation. Given the number of comparisons among secondary outcomes, we applied the Benjamini-Hochberg procedure to adjust *P* values, controlling for the false discovery rate at 0.05. Adjusted *P* values below this threshold were considered statistically significant, and R statistical program version 4.4.2 was used for all statistical calculations.

## RESULTS

Between 2011 and 2023, 1889 patients with pancreatic cancer underwent radical surgery. The mean age was 67.4 (SD, 9.1) years, and 53.5% were male [Table 1]. Most patients had an AJCC stage II tumor (65.3%), and pancreaticoduodenectomy (Whipple's procedure) was the most common operation (67.0%). The mean duration of hospitalization was 13.6 days (median: 10.0 days), whereas the mortality was 0.6% (95% CI, 0.3%–1.0%) at 30 days and 2.4% (95% CI, 1.7%–3.2%) at 90 days. The mean follow-up time was 31.2 months (range: 1–145 months). Preoperative EUS-B was performed in a minority of the patients (*n* = 243, 12.9%). The groups were mostly similar, but a larger proportion of patients in the EUS-B group received NACT (20.2% *vs.* 7.2%; *P* < 0.001).

**Table 1:** Patient demographics in the overall unmatched cohort.

	No EUS-B (*N* = 1646)	EUS-B (*N* = 243)	*P*
Sex			
Male	872 (53.0%)	138 (56.8%)	0.994
Female	774 (47.0%)	105 (43.2%)	
Age, yr			
Mean (SD)	67.4 (9.25)	67.1 (8.43)	1.000
Median [Min, Max]	69.0 [11.0, 87.0]	68.0 [40.0, 81.0]	
CCI			
Mean (SD)	1.35 (1.83)	1.23 (1.80)	1.000
Median [Min, Max]	1.00 [0, 12.0]	1.00 [0, 11.0]	
Histopathology			
Adenocarcinoma	1568 (95.3%)	234 (96.3%)	1.000
Mucinous adenocarcinoma	15 (0.9%)	2 (0.8%)	
IPMN-associated carcinoma	5 (0.3%)	1 (0.4%)	
Signet ring cell carcinoma	3 (0.2%)	0 (0%)	
Adenosquamous carcinoma	46 (2.8%)	5 (2.1%)	
Acinic cell carcinoma	6 (0.4%)	1 (0.4%)	
Other	3 (0.2%)	0 (0%)	
AJCC stage			
1	247 (15.0%)	39 (16.0%)	1.000
2	1078 (65.5%)	155 (63.8%)	
3	321 (19.5%)	49 (20.2%)	
Type of resection			
Distal pancreatectomy	256 (15.6%)	52 (21.4%)	0.164
Total pancreatectomy	285 (17.3%)	30 (12.3%)	
Pancreaticoduodenectomy	1105 (67.1%)	161 (66.3%)	
Arterial resection			
No	1627 (98.8%)	239 (98.4%)	1.000
Yes	19 (1.2%)	4 (1.6%)	
Neoadjuvant therapy			
No	1527 (92.8%)	194 (79.8%)	<0.001
Yes	119 (7.2%)	49 (20.2%)	
Adjuvant therapy			
No	532 (32.3%)	79 (32.5%)	1.000
Yes	1114 (67.7%)	164 (67.5%)	

AJCC: American Joint Committee on Cancer; CCI: Charlson comorbidity index; EUS-B: EUS–guided biopsy; IPMN: Intraductal papillary mucinous neoplasm.

### Overall survival in unselected cohort

**Figure 1:**
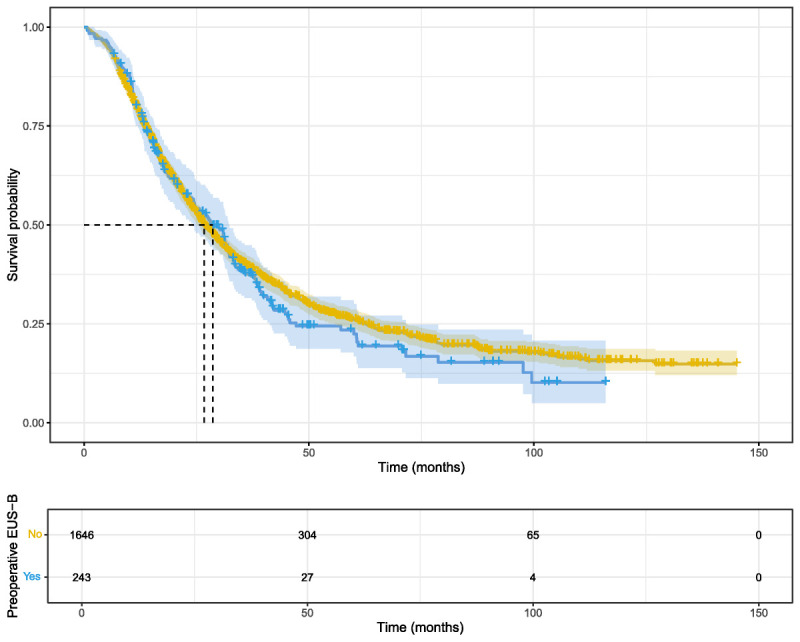
Kaplan-Meier plot of overall survival in the overall cohort. Survival probabilities for patients who underwent EUS-B (blue) are compared with those who did not (yellow). Median survival time is marked with a dashed line.

In the unselected cohort, the median overall survival was 28.5 months in the EUS-B group (95% CI, 24.3–32.3) compared to 26.7 months in the non–EUS-B group (95% CI, 25.5–28.9). In the univariable Cox model, preoperative EUS-B was not associated with overall survival (HR, 1.07; 95% CI, 0.91–1.26; *P* = 0.411). The Kaplan-Meier plot showed similar survival probabilities throughout the follow-up period [Figure 1]. Associations were further evaluated within subgroups of patients defined by the type of resection (pancreaticoduodenectomy, distal, and total pancreatectomy), AJCC stage, sex, age (<70 *vs.* ≥70 years), and adjuvant therapy (SDC, Table 1, http://links.lww.com/ENUS/A387). There was no evidence of an association between EUS-B and inferior survival in any of the subgroups.

### Overall survival in the propensity score–matched cohort

**Figure 2:**
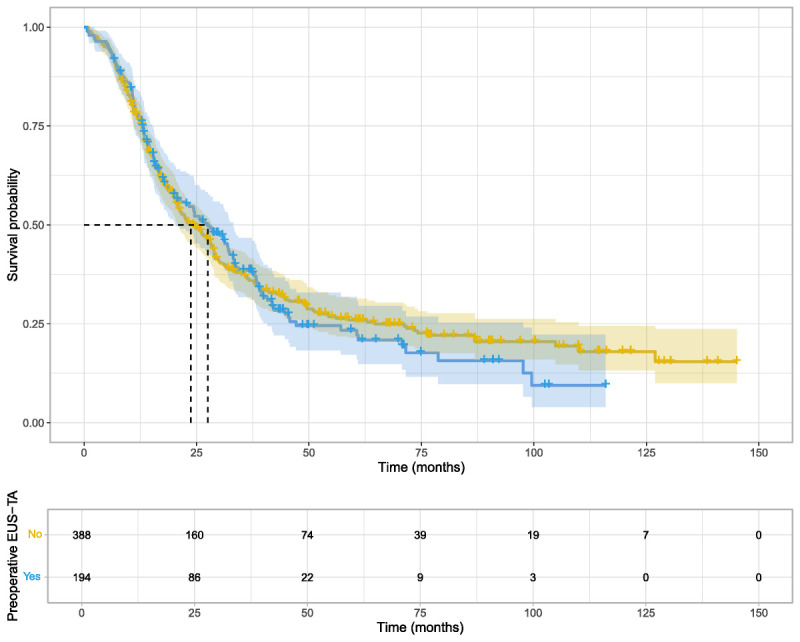
Kaplan-Meier plot of overall survival in the propensity score–matched cohort. The blue line represents patients who underwent EUS-B, whereas the yellow line depicts the survival probabilities of those who did not.

Of 243 EUS-B cases, 194 were matched to 388 controls in a 1:2 ratio, yielding 582 patients in the matched cohort. The remaining 49 cases received NACT and were thus excluded. The standardized mean differences were minimal, with the highest observed value of 0.03, indicating well-matched data [SDC, Figure 1, http://links.lww.com/ENUS/A387]. The two groups exhibited nearly identical baseline characteristics [Table 2]. The median survival time was 28.6 months in the EUS-B group (95% CI, 22.7–33.4) and 24.7 months in the non–EUS-B group (95% CI, 21.2–28.9). Cox regression analysis did not reveal any associations between EUS-B and survival (HR, 1.04; 95% CI, 0.84–1.29; *P* = 0.691), and the Kaplan-Meier plot demonstrated similar survival between the two groups [Figure 2]. Although established survival predictors such as age, CCI, and tumor stage showed clear effects on survival in the multivariable model [Table 3], EUS-B was not associated with any difference in survival (HR, 1.04; 95% CI, 0.84–1.28; *P* = 1.000). Finally, subgroups were formed based on the type of resection, AJCC stage, sex, age (<70 *vs.* ≥70 years), and adjuvant therapy, but EUS-B was not associated with overall survival in any of these subgroups [SDC, Table 1, http://links.lww.com/ENUS/A387].

**Table 2:** Patient demographics in the smaller propensity score–matched cohort.

	No EUS-B (*N* = 388)	EUS-B (*N* = 194)	*P*
Sex			
Male	226 (58.2%)	110 (56.7%)	1.000
Female	162 (41.8%)	84 (43.3%)	
Age, yr			
Mean (SD)	67.5 (9.08)	67.7 (8.51)	1.000
Median [Min, Max]	69.0 [11.0, 84.0]	69.0 [40.0, 81.0]	
CCI			
Mean (SD)	1.16 (1.56)	1.37 (1.93)	1.000
Median [Min, Max]	1.00 [0, 9.00]	1.00 [0, 11.0]	
Histopathology			
Adenocarcinoma	367 (94.6%)	187 (96.4%)	1.000
Mucinous adenocarcinoma	3 (0.8%)	2 (1.0%)	
IPMN-associated carcinoma	5 (1.3%)	1 (0.5%)	
Signet ring cell carcinoma	1 (0.3%)	0 (0%)	
Adenosquamous carcinoma	8 (2.1%)	3 (1.5%)	
Acinic cell carcinoma	3 (0.8%)	1 (0.5%)	
Other	1 (0.3%)	0 (0%)	
AJCC stage			
1	48 (12.4%)	27 (13.9%)	1.000
2	259 (66.8%)	129 (66.5%)	
3	81 (20.9%)	38 (19.6%)	
Type of resection			
Distal pancreatectomy	65 (16.8%)	43 (22.2%)	0.978
Total pancreatectomy	45 (11.6%)	20 (10.3%)	
Pancreaticoduodenectomy	278 (71.6%)	131 (67.5%)	
Arterial resection			
No	388 (100%)	194 (100%)	1.000
Yes	0 (0%)	0 (0%)	
Neoadjuvant therapy			
No	388 (100%)	194 (100%)	1.000
Yes	0 (0%)	0 (0%)	
Adjuvant therapy			
No	115 (29.6%)	63 (32.5%)	1.000
Yes	273 (70.4%)	131 (67.5%)	

AJCC: American Joint Committee on Cancer; CCI: Charlson comorbidity index; EUS-B: EUS–guided biopsy; IPMN: Intraductal papillary mucinous neoplasm.

**Table 3:** Univariable and multivariable survival analyses in the propensity score–matched cohort.

	HR (95% CI)	*P*
**Univariable Cox regression**		
EUS-B	1.04 (0.84–1.29)	0.411
**Multivariable Cox regression**		
EUS-B	1.04 (0.84–1.28)	0.691
Age	1.02 (1.01–1.03)	0.016
Sex (Ref: Male)	0.84 (0.69–1.03)	0.501
CCI	1.09 (1.03–1.15)	0.016
AJCC tumor stage (Ref: Stage I)		
Stage II	2.35 (1.63–3.39)	<0.001
Stage III	3.02 (1.99–4.58)	<0.001
Adjuvant therapy (Ref: No)	0.83 (0.64–1.07)	0.687

AJCC: American Joint Committee on Cancer; CCI: Charlson comorbidity index; EUS-B: EUS–guided tissue acquisition.

## DISCUSSION

Preoperative EUS-guided biopsy had no impact on overall survival in this nationwide, retrospective study. No associations were found in the unselected cohort (HR, 1.07; 95% CI, 0.91–1.26; *P* = 0.411) or in the propensity score–matched subcohort (HR, 1.04; 95% CI, 0.84–1.29; *P* = 0.691).

Survival in cancer is influenced by a complex interplay of multifactorial determinants, including tumor biology, patient demographics, comorbidities, genetic predispositions, and treatment modalities. Although patient-related factors such as age, sex, genetic predisposition, and baseline health status are generally considered fixed, therapeutic strategies, including the choice and timing of treatment, should be tailored to provide the maximum survival benefit. Arguments against preoperative EUS-B include the risk of tumor cell seeding along the needle tract, potential adverse events, and the possibility of false-negative biopsies, all of which can delay treatment initiation and negatively impact survival. Tumor recurrence along the needle tract is considered rare, and although the risk is lower in EUS-guided biopsies compared to percutaneous methods, there are some published reports.^[2,7–9]^ A meta-analysis published in 2022, which included 10 studies and 13,238 patients, estimated this risk to be 0.3% (95% CI, 0.2%–0.4%). However, the results should be interpreted with caution, as one study contributed over 94% of the total weight.^[10]^ Similar occurrences of metachronous peritoneal carcinomatosis were observed in both groups, suggesting that preoperative EUS-B did not increase the risk of peritoneal seeding. However, most recurrences occurred in the remnant gastric wall, most likely in patients who underwent distal pancreatectomy. EUS-B is generally regarded as safe, and in a retrospective Danish single-center study of 723 patients who underwent pancreatic EUS-B for the entire spectrum of indications during 2015–2020, adverse events requiring intervention occurred only in 0.2% (*n* = 2) in the 7 day-period following the procedure.^[11]^ These events included one case of acute necrotizing pancreatitis and one case of duodenal bleeding. Conservatively treated minor adverse events (4.7%) were consistent with previous publications reporting complication rates of 3–6%.^[12–15]^

Several studies have examined the association between EUS-B and overall survival but found no evidence of inferior survival in the EUS-B group.^[5,7,16–18]^ Ngamruengphong et al. even observed a survival benefit from EUS-B, reporting longer median survival compared to non–EUS-B (22 *vs.*15 months, *P* = 0.03) and longer cancer-specific survival (24 *vs.* 18 months, *P* = 0.04) in a large retrospective cohort of 2034 patients.^[19]^ However, this is most likely due to selection bias, as patients with smaller tumors, which can be difficult to diagnose radiologically, may be more likely to undergo EUS-B. In another large retrospective study, Park et al. examined 528 patients who underwent distal pancreatectomy.^[12]^ The EUS-B and non–EUS-B groups had similar survival (28.9 *vs*. 25.1 months, *P* = 0.10) and recurrence-free survival (12.4 *vs*. 12.1 months, *P* = 0.69). The only propensity score–matched study published to date included 153 patients.^[8]^ The study did not find any difference in overall survival; however, propensity score matching did not account for neo- or adjuvant therapy, leading to unequal distribution between the groups (22.2% *vs.* 2.4% for neoadjuvant therapy in the EUS-B compared to the non–EUS-B group; *P* < 0.001). Given that the estimated occurrence of tumor cell seeding is rare, small studies may not have sufficient power to detect potential survival differences. Furthermore, EUS-B has undergone significant technical changes over the past few decades, transitioning from aspiration needles (FNA) to tissue-core needles (FNB).^[20]^ None of the previously mentioned studies reported on the needle size or type used, nor did the DPCG database provide this information, which is why it is yet to be determined whether any of the different needle designs might increase the risk of tumor cell seeding along the needle tract.

The results of our study are consistent with most previous studies, which reported no association between EUS-B and survival. We performed exploratory analyses within distinct subgroups, adjusting for age, sex, AJCC stage, type of resection (to account for tumor location), and adjuvant therapy, but found no evidence that EUS-B is associated with reduced survival in patients undergoing distal pancreatectomy, as previously theorized. The strengths of our study include nationwide “real-world” data with a large number of patients and high-quality, reliable data sources, including mortality data. Moreover, the use of preoperative EUS-B at tertiary treatment centers varies across Denmark, providing a form of pseudo-randomization at the national level based on geography. To further minimize the risk of bias, propensity score matching was performed, and the two groups had nearly identical distributions of the known factors associated with survival. Propensity score matching is a robust method for reducing selection bias and simulating randomization, improving the validity of causal inferences, and minimizing confounding effects that could otherwise distort the study results. Nonetheless, this study has some limitations: its retrospective nature and long time span may have introduced heterogeneity due to differences in care and biopsy needle types. Additionally, the DPCG database lacks information on recurrence-free survival and recurrence type, preventing assessment of effects of EUS-B on disease-free survival, which may not translate into an overall survival difference. Most patients in our cohort did not undergo EUS-B, potentially reducing statistical power and limiting generalizability due to relative underrepresentation. Finally, the cohort included only patients undergoing surgery, so the results cannot be extrapolated to the broader pancreatic cancer population or to all patients undergoing EUS-B.

## CONCLUSION

Preoperative EUS-guided biopsy did not affect overall survival of patients with pancreatic cancer in this large, nationwide retrospective study.

## Supplementary information

Supplementary materials are only available at the official website of the journal (www.eusjournal.com).

## Author Contributions

Bojan Kovacevic designed and conducted the research, provided essential materials, analyzed data, performed statistical analysis, wrote the paper, and had primary responsibility for the final content. Claus Fristrup designed and conducted the research, provided essential materials, analyzed data, and wrote the paper. Carsten P. Hansen conducted the research, provided essential materials, and wrote the paper. Michael B. Mortensen conducted the research, provided essential materials, and wrote the paper. Frank Mortensen conducted the research, provided essential materials, and wrote the paper. Jakob Kirkegård conducted the research, provided essential materials, analyzed data, performed statistical analysis, and wrote the paper. Mogens T. Stender conducted the research, provided essential materials, and wrote the paper. Sönke Detlefsen conducted the research, provided essential materials, and wrote the paper. Peter Vilmann designed the research, provided essential materials, wrote the paper, and had primary responsibility for the final content. All authors have read and approved the final manuscript.

## Source of Funding

This study was supported by a research grant from Copenhagen University Hospital Herlev and Gentofte, awarded to Bojan Kovacevic.

## Ethical Approval

The study was approved by The Regional Committee on Health Research Ethics for Southern Denmark (no. 23/18843).

## Informed consent

Not applicable.

## Conflicts of Interest

The authors declare that they have no known competing financial interests or personal relationships that could have appeared to influence the work reported in this paper.

## Data Availability Statement

The data that support the findings of this study are not publicly available due to privacy and ethical restrictions and are only accessible upon reasonable request to the corresponding author.

## References

[1] Wiersema MJ Vilmann P Giovannini M Chang KJ Wiersema LM. Endosonography-guided fine-needle aspiration biopsy: diagnostic accuracy and complication assessment. *Gastroenterology* 1997;112(4):1087–1095. doi:10.1016/s0016-5085(97)70164-1. 9097990

[2] Paquin SC Gariepy G Lepanto L Bourdages R Raymond G Sahai AV. A first report of tumor seeding because of EUS-guided FNA of a pancreatic adenocarcinoma. *Gastrointest Endosc* 2005;61(4):610–611. doi:10.1016/s0016-5107(05)00082-9. 15812422

[3] Conroy T Pfeiffer P Vilgrain V, . Pancreatic cancer: ESMO clinical practice guideline for diagnosis, treatment and follow-up. *Ann Oncol* 2023;34(11):987–1002. doi:10.1016/j.annonc.2023.08.009. 37678671

[4] Okusaka T Nakamura M Yoshida M, . Clinical practice guidelines for pancreatic cancer 2022 from the Japan Pancreas Society: a synopsis. *Int J Clin Oncol* 2023;28(4):493–511. doi:10.1007/s10147-023-02317-x. 36920680 PMC10066137

[5] Beane JD House MG Cote GA, . Outcomes after preoperative endoscopic ultrasonography and biopsy in patients undergoing distal pan-createctomy. *Surgery* 2011;150(4):844–853. doi:10.1016/j.surg.2011.07.068. 22000199

[6] Ngamruengphong S Xu C Woodward TA, . Risk of gastric or peritoneal recurrence, and long-term outcomes, following pancreatic cancer resection with preoperative endosonographically guided fine needle aspiration. *Endoscopy* 2013;45(8):619–626. doi:10.1055/s-0033-1344216. 23881804

[7] Yane K Kuwatani M Yoshida M, . Non-negligible rate of needle tract seeding after endoscopic ultrasound–guided fine-needle aspiration for patients undergoing distal pancreatectomy for pancreatic cancer. *Dig Endosc* 2020;32(5):801–811. doi:10.1111/den.13615. 31876309

[8] Kitano M Yoshida M Ashida R, . Needle tract seeding after endoscopic ultrasound–guided tissue acquisition of pancreatic tumors: a nationwide survey in Japan [published online May 3, 2022]. *Dig Endosc* . doi:10.1111/den.14346. 35502924

[9] Terasawa H Matsumoto K Tanaka T, . Cysts or necrotic components in pancreatic ductal adenocarcinoma is associated with the risk of EUS-FNA/B complications including needle tract seeding. *Pancreatology* 2023;23(8):988–995. doi:10.1016/j.pan.2023.10.018. 37951728

[10] Facciorusso A Crino SF Gkolfakis P, . Needle tract seeding after endoscopic ultrasound tissue acquisition of pancreatic lesions: a systematic review and meta-analysis. *Diagnostics (Basel)* 2022;12(9):2113. doi:10.3390/diagnostics12092113. 36140514 PMC9498098

[11] Thomsen MM Larsen MH Di Caterino T Hedegaard Jensen G Mortensen MB Detlefsen S. Accuracy and clinical outcomes of pancreatic EUS-guided fine-needle biopsy in a consecutive series of 852 specimens. *Endosc Ultrasound*. 2022;11(4):306–318. doi:10.4103/EUS-D-21-00180. 35708361 PMC9526106

[12] Jovani M Abidi WM Lee LS. Novel fork-tip needles versus standard needles for EUS-guided tissue acquisition from solid masses of the upper GI tract: a matched cohort study. *Scand J Gastroenterol* 2017;52(6-7):784–787. doi:10.1080/00365521.2017.1306879. 28355953

[13] Di Leo M Crinò SF Bernardoni L, . EUS-guided core biopsies of pancreatic solid masses using a new fork-tip needle: a multicenter prospective study. *Dig Liver Dis* 2019;51(9):1275–1280. doi:10.1016/j.dld.2019.03.025. 31010744

[14] Fitzpatrick MJ Hernandez-Barco YG Krishnan K Brugge W Casey B Pitman MB. Diagnostic yield of the SharkCore EUS-guided fine-needle biopsy. *J Am Soc Cytopathol* 2019;8(4):212–219. doi:10.1016/j.jasc.2019.03.001. 31076375

[15] DiMaio CJ Kolb JM Benias PC, . Initial experience with a novel EUS-guided core biopsy needle (SharkCore): results of a large North American multicenter study. *Endosc Int Open* 2016;4(9):E974–E979. doi:10.1055/s-0042-112581. 27652304 PMC5025313

[16] Maruo M Ikeura T Takaori A, . Impact of endoscopic ultrasound–guided tissue acquisition on prognosis and peritoneal lavage cytology in resectable or borderline resectable pancreatic ductal adenocarcinoma. *Pancreatology* 2024;24(5):787–795. doi:10.1016/j.pan.2024.06.001. 38871559

[17] Park JS Lee JH Song TJ, . The impact of preoperative EUS-FNA for distal resectable pancreatic cancer: is it really effective enough to take risks? *Surg Endosc* 2022;36(5):3192–3199. doi:10.1007/s00464-021-08627-3. 34254183

[18] Kim SH Woo YS Lee KH, . Preoperative EUS-guided FNA: effects on peritoneal recurrence and survival in patients with pancreatic cancer. *Gastrointest Endosc* 2018;88(6):926–934. doi:10.1016/j.gie.2018.06.024. 29981302

[19] Ngamruengphong S Swanson KM Shah ND Wallace MB. Preoperative endoscopic ultrasound–guided fine needle aspiration does not impair survival of patients with resected pancreatic cancer. *Gut* 2015;64(7):1105–1110. doi:10.1136/gutjnl-2014-307475. 25575893

[20] Kovacevic B Vilmann P. EUS tissue acquisition: from A to B. *Endosc Ultrasound* 2020;9(4):225–231. doi:10.4103/eus.eus_21_20. 32655082 PMC7528999

